# Effects of Different Levels of Carbohydrates on Growth Performance, Hepatic and Intestinal Health, and Intestinal Microflora of Juvenile Pikeperch (*Sander lucioperca*)

**DOI:** 10.1155/2024/8450154

**Published:** 2024-08-09

**Authors:** Jie Zhao, Yang Liu, Zhipeng Sun, Liansheng Wang, Ze Fan, Yadan Pan, Jiamin Gao, Cuiyun Lu, Xianhu Zheng

**Affiliations:** ^1^ National and Local Joint Engineering Laboratory for Freshwater Fish Breeding Heilongjiang River Fisheries Research Institute Chinese Academy of Fishery Sciences, Harbin 150070, China; ^2^ College of Fish and Life Science Shanghai Ocean University, Shanghai 201306, China; ^3^ College of Fisheries Tianjin Agricultural University, Tianjin 300392, China

## Abstract

Pikeperch (*Sander lucioperca*) is a species with great potential for aquaculture in Eurasian countries, while feed costs limit the scale of pikeperch farming. Adding carbohydrates to the feed as an energy source is a viable approach to reduce costs and to improve the culture status of pikeperch. In this study, in order to determine the optimal carbohydrate requirement of pikeperch, three tapioca starch (8%, 10%, and 12%) added feeds were produced with isonitrogenous (51%) and isolipidic (11%). For 8 weeks, body weight was 1.20 ± 0.01 g, pikeperch were manually fed the trio of experimental diets until they seemed fully satisfied. The finding revealed that pikeperch can utilize dietary carbohydrate, but excessive dietary carbohydrate will adversely affect the growth performance. The growth and survival rate were decreased in pikeperch in S12 (*P* < 0.05). The *α*-amylase activity of S12 reduced in the intestine and lipid deposition was observed in the liver compared with the S8. In addition, proinflammatory cytokines, interleukin 1 beta (*il1-β*), interleukin 8 (*il8*), and tumor necrosis factor beta (*tnf-β*), in the liver and intestine elevate and anti-inflammatory cytokines, interleukin 10 (*il10*) and transforming growth factor beta (*tgf-β*), decrease with increasing dietary carbohydrate levels. Hepatic and intestinal antioxidant capacity were also adversely affected, with S12 significantly increasing malondialdehyde (MDA) contents and decreasing glutathione (GSH) and total antioxidant capacity (T-AOC) (*P* < 0.05). The intestinal barrier function is also damaged, the height and width of intestinal villi decreased, and the expression of *occludin-a*, *occludin-b*, and zonula occludens-2 (*zo-2*) genes was decreased. Elevated levels of starch intake led to harm to gut microflora, reducing bacterial populations, simultaneously boosting the presence of detrimental bacteria (Proteobacteria, Actinobacteriota, Achromobacter, and Rhodococcus) and diminishing the beneficial bacteria (Firmicutes). In conclusion, moderate addition of starch as an energy source can reduce feed costs; however, over addition can bring about organism damage and is recommended to be added at less than 10%.

## 1. Introduction

Pikeperch (*Sander lucioperca*) has long been considered a potential freshwater carnivorous fish because of its tender flesh, rapid growth, and high commercial value, especially in Europe [1, 2, 3, 4, 5]. However, there are still few studies on the basic nutritional needs of pikeperch, and although the existing studies have given the requirement for protein is 47.0%–57.7% and lipid is 10.0%–17.0% [6, 7, 8, 9]. The specific requirements of carbohydrates have not yet been known. Determining optimal dietary carbohydrate requirements will be particularly important for large-scale pikeperch farming.

Carbohydrates are rich in resources and low cost, which can be used as the most economical source of energy for animals. Feed-added carbohydrates can improve the utilization rate of protein, prevent protein from being metabolized as energy substances, and improve the production performance of animals [10]. At the same time, nitrogen emissions will be greatly reduced, reducing environmental pollution [11]. Many studies have implied that there existed differences in the requirements on carbohydrates among different fishes. Omnivorous or herbivorous fish can make good use of carbohydrates in their diet, and the carbohydrate content in some fish diets can even be as high as 40% [12], while carnivorous fish do use carbohydrates is generally lower level than omnivorous and herbivorous fish [13, 14, 15]. Moderate carbohydrate levels lead to better growth performance and can save protein use, but when feed carbohydrate levels are too high, the bioavailability of fish also decreases [16, 17]. Additionally, an overabundance of dietary starch may adversely affect fish health by disrupting metabolism and triggering inflammation, such as hepatic steatosis [18], glycogen deposition [19], and inflammation response [20].

Prior research has shown that pikeperch (51.1 ± 2.4 g) experience better growth when fed with diets containing 15% and 20% carbohydrates [6]. However, the optimal carbohydrate requirement for juvenile pikeperch was not indicated. Three diets were designed with varying amounts of carbohydrates to determine the optimal amount for adding to the feed of pikeperch, considering growth performance, hepatic and intestinal health, and intestinal microflora. To assess how different carbohydrate concentrations affect pikeperch, the following indicators were examined: growth performance and body composition, histology and enzyme capacity in the liver and intestine, mRNA expression of immune-related genes, tight junction protein genes, mammalian target of rapamycin (mTOR) pathway genes, and analysis of the intestinal microflora.

The study aimed to explore how varying carbohydrate levels in the diet impact the growth performance, hepatic and intestinal health, and intestinal microflora, and to provide a reference of the carbohydrate needs of juvenile pikeperch.

## 2. Materials and Methods

### 2.1. Animal Ethics

The Heilongjiang River Fisheries Institute's Committee for the Welfare and Ethics of Laboratory Animals (CAFS) sanctioned every animal-related procedure in this research. Our approach adhered to the established institutional standards for laboratory animal care and usage.

### 2.2. Test Diets

Based on previous studies [6, 8, 9], three kinds of feeds were prepared, with protein content of 51% and lipid content of 11% as the base feed, and carbohydrate levels of 15%, 17%, and 19% were designated. Since starch is the main determinant of carbohydrate levels, adding different amounts of tapioca starch (ungelatinized starch) to make feeds with 8%, 10%, and 12% starch content (Table 1), the groupings in this paper are named after starch. All ingredients were passed through a 60-mech particle size. The protein source was fishmeal, poultry meal, soy protein concentrate, and corn gluten meal. The lipid source was fish oil and lecithin oil. Vitamins and minerals were provided by premix-V + M. Subsequently, every nutrient was meticulously blended using the progressive enlargement technique. Then lipid and water were added to prefeeds and mixed well after granulation mechanism into 1.5-mm particle size pellet feed. Feed was dried until moisture was less than 5% and stored at −20°C.

### 2.3. Experiment Fish and Feeding Management

A total of 1,350 juvenile pikeperch (1.20 ± 0.01 g) were evenly and randomly divided into nine tanks (500 L) at the Hulan experiment station, Heilongjiang River Fisheries Research Institute. In groups of three tanks, fishes were fed diets with 8%, 10%, and 12% starch content for 8 weeks, respectively. The control water flow was 200 L/hr, water temperature was 23 ± 1°C, pH was 7.5–7.9, dissolved oxygen was >7 mg/L, and ammonia nitrogen was <0.2 mg/L. These fishes were manually fed twice a day (7:00 and 17:00) until obvious satiety occurs. The photoperiod was naturally scattered light. Regular daily cleaning of the fish tank was conducted to eliminate fecal waste.

### 2.4. Sample Collection

At the conclusion of the feeding trial, 15 fishes were randomly chosen to undergo anesthesia with tricaine methane sulfonate (MS-222, 200 mg/L; Sigma-Aldrich, St. Louis, USA). Their weight and length were measured after anesthesia. The liver and intestines of the fish were collected on an ice tray and weighed to calculate the hepatosomatic index and viscerosomatic index. The samples of liver and intestine were then divided into two parts and stored in liquid nitrogen and 30% formalin solution. Then, samples of fecal were gathered and preserved in liquid nitrogen for intestinal microflora analysis. Liver and whole intestine samples in liquid nitrogen were used for assaying gene expression and enzyme activity, while the midgut samples in formalin solution were used for histology. Additionally, six fishes were taken from each tank and stored in a freezer at −20°C for analysis of the composition of whole fish.

### 2.5. Chemical Composition of Whole Fish

Analysis of the chemical composition of the diet and fish samples was performed using standard AOAC [24] methods. Refer to Table 1 of Supplementary Materials I for further information.

### 2.6. Analysis of Intestinal Microflora

A commercial DNA kit (MP FastDNA Spin Kit for soil, MP Biomedicals, Irvine, CA, USA) was employed to extract the complete DNA (*n* = 5) of intestinal bacteria, with the extracted DNA being mass-detected using 1% agarose gel electrophoresis. Amplification of the V3-V4 highly variable segments of the bacterial 16S rRNA gene was achieved through the Thermocycler PCR system (GeneAmp 9700, ABI, USA), employing bacterial universal primers 338F and 806R. The amplification conditions were those reported in the previous study [25], and postpurification, sequencing of all amplicons was conducted using the Illumina MiSeq system (Illumina, San Diego, CA, USA).

Biological analysis of the intestinal microflora, first to clip the original data sequence, using Majorbio Cloud Platform (http://www.majorbio.com) based on previously reported methods [26, 27]. Then, according to the rules in Quantitative Insights into Microbial Ecology (QIIME, version 1.8.0) [28], qualified reads were assigned to individual OTUs, with members in each OTU having 97% similarity. Based on bacterial operational taxonomic units(OTUs), Sobs, Chao, ACE, Coverage Simpson, Shannon, and other parameters were used to evaluate the *α*-diversity. Analysis of *β*-diversity was conducted through principal component analysis (PCA) utilizing the ape package. The species classification database was silva138/16s_bacteria. Clustering was done using the USEARCH11-uparse algorithm. Conducted an analysis of the Veen graph to pinpoint distinct and common OTUs among different groups. Variations in the diversity of intestinal flora among different groups were examined at both phylum and genus levels. To pinpoint varying bacterial species among groups, the Python LEfSe package [29] was employed for conducting linear discriminant analysis effect size (LEfSe) analysis.

### 2.7. Antioxidant Enzyme and Digestive Enzyme Activity

In this experiment, the antioxidant enzyme and digestive enzyme activity of liver and whole intestine were assayed by commercial kits (Jiancheng Biotech. Co., Nanjing, China) through the colorimetric method. Refer to *Supplementary table 1* for further information.

### 2.8. Gene Expression

A random selection of six samples per group was made for RNA extraction using TRIzol Reagent (Invitrogen, Carlsbad, CA, USA). Utilizing the PrimeScript™ RT reagent Kit and gDNA Eraser (Perfect Real Time, Takara, Dalian, China), the RNA extracted underwent reverse transcription into cDNA, and the cDNA was preserved at −80°C for subsequent examination. The RT-qPCR process utilized Applied Biosystems 7500 (ABI, Waltham, MA, USA), employed TB Green Premix Ex Taq II (Tli RnaseH Plus, Takara, Dalian, China) as the reagent, and maintained a reaction volume of 20 *μ*L. The expression levels of specific gene mRNA were ascertained through the threshold cycle (2^−*ΔΔCt*^) technique [30]. The reference gene was glyceraldehyde-3-phosphate dehydrogenase (*gapdh*). For a more accurate evaluation of carbohydrates' impact on pikeperch growth, the expression of specific genes were analyzed, include AKT serine/threonine kinase 1 (*akt1*), mechanistic target of rapamycin kinase (*mtor*), ribosomal protein S6 kinase B1 (*rps6k1*), and eukaryotic initiation factor 4E binding protein (*eif4ebp*). For a more accurate evaluation of carbohydrates' impact on pikeperch health, the expression of specific genes was analyzed, including interleukin 1 beta (*il1-β*), interleukin 8 (*il8*), interleukin 10 (*il10*), tumor necrosis factor beta (*tnf-β*), transforming growth factor beta (*tgf-β*), nuclear factor *κ*-B P65 (*nf-κb p65*), *occludin-a*, *occludin-b*, zonula occludens-2 (*zo-2*), and *claudin-15a*. Refer to *Supplementary tables 1 and 2* for further information.

### 2.9. Histology of the Liver and Midgut

Six liver and intestinal tissues were randomly selected from each group and fixed in 30% formalin solution. All the slices of tissue samples were manufactured by Servicebio Co., Wuhan, China. For each segment, nine unharmed villus and crypts were chosen, and measurements of the villus's width, height, and the thickness of the intestinal wall were conducted using Image Pro Plus software.

### 2.10. Statistical Analysis

The SPSS 19.0 software was used to perform one-way ANOVA and Duncan's multiple range test analysis on the experimental data, and the mean difference between the groups was analyzed. The difference was significant at a level of *P* < 0.05.

## 3. Results

### 3.1. Growth and Feed Utilization

The survival rate (SR) of S12 was significantly less compared to S8 and S10 (*P* < 0.05), whereas S8 and S10 showed no notable disparity (*P* > 0.05). Final body weight (FBW), weight gain rate (WGR), specific growth rate (SGR), and condition factor (CF) showed a gradual decreasing trend with increasing dietary carbohydrate. FBW, WGR, and CF in the two groups of S8 and S10 were significantly greater compared to S12 (*P* < 0.05). Meanwhile, feed conversion rate (FCR) of S8 was significantly lower than that of S12 (*P* < 0.05), and there was no significant disparity between S8 and S10 (*P* > 0.05). No notable variance was observed in viscerosomatic index (VSI) across the treatment cohorts, yet hepatosomatic index (HSI) levels were considerably reduced in S8 and S10 compared to S12 (*P* < 0.05; Table 2).

(1) $$\begin{array}{c} \text{Survival rate }\left(\text{SR},\text{ \% }\right)=100\times \left(\text{Final number of fish}/\text{initial number of fish}\right), \end{array}$$

(2) $$\begin{array}{c} \text{Weight gain rate }\left(\text{WGR},\text{ \% }\right)=100\times \left(\text{FBW}-\text{IBW}\right)/\text{IBW}, \end{array}$$

(3) $$\begin{array}{c} \text{Specific growth rate }\left(\text{SGR},\text{ \%}/\text{day}\right)=100\times \left(\text{Ln }\left(\text{FBW}\right)-\text{ Ln }\left(\text{IBW}\right)\right)/\text{days}, \end{array}$$

(4) $$\begin{array}{c} \text{Feed conversion rate }\left(\text{FCR}\right)=\text{Feed intake}/\left(\text{FBW}-\text{IBW}\right), \end{array}$$

(5) $$\begin{array}{c} \text{Condition factor }\left(\text{CF},g/{\text{cm}}^{3}\right)=100\times \text{Body weight}/{\left(\text{body length}\right)}^{3}, \end{array}$$

(6) $$\begin{array}{c} \text{Viscerosomatic index }\left(\text{VSI},\text{ \% }\right)=100\times \text{Viscera wet weight}/\text{IBW}, \end{array}$$

(7) $$\begin{array}{c} \text{Hepatosomatic index }\left(\text{HSI},\text{ \% }\right)=100\times \text{Liver wet weight}/\text{IBW}. \end{array}$$

### 3.2. Chemical Composition of Whole Fish

There were no significant variances in moisture and ash content among treatments. The crude protein and crude lipid of whole fish in S12 were significantly less compared to other treatments (*P* < 0.05), with no significant disparity between S8 and S10 (Table 3).

### 3.3. Hepatic and Intestinal Histology

**Figure 1:**
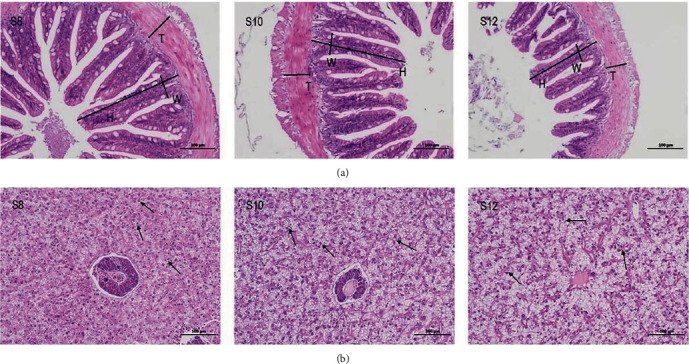
(a and b) Effect of the dietary carbohydrate on midgut and liver histology of pikeperch. H, villus height; W, villus width; and T, intestinal wall thickness.

It was distinctly observed that intestinal villus height decreased with increasing dietary carbohydrate levels (Table 4 and Figure 1(a)). Intestinal villus width and intestinal wall thickness were significantly lower in S12 compared to S8 (*P* < 0.05), but there was no significant disparity between S8 and S10. Furthermore, the size of hepatocytes also increased, the nucleus shifted towards the edge, and the cells exhibited signs of vacuolation with increasing dietary carbohydrate (Figure 1(b)).

### 3.4. Digestive Enzyme Activity of Intestine

**Figure 2:**
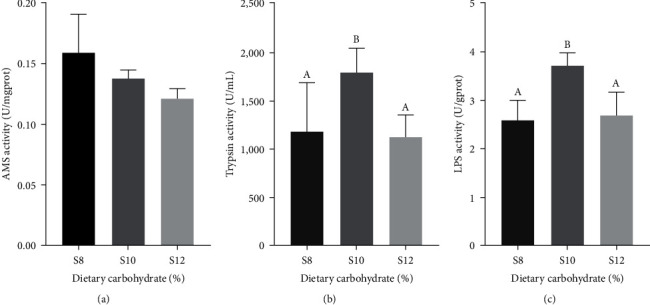
(a–c) Activity of digestive enzyme in intestine of pikeperch fed different dietary carbohydrate levels diets.

Analysis of intestinal digestive enzyme activities revealed that *α*-amylase activity showed a gradual decrease with an increasing dietary carbohydrate level, but there was no significant disparity (*P* > 0.05). Trypsin and lipase both achieved maximum values at S10 and were significantly greater compared to S8 and S12 (*P* < 0.05; Figure 2).

### 3.5. Activity of Antioxidant Enzymes in Liver and Intestine

**Figure 3:**
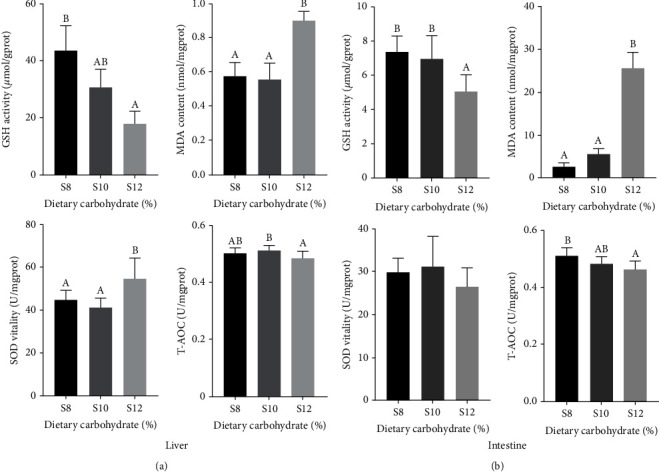
Activity of antioxidant enzyme in liver (a) and intestine (b) of pikeperch fed different dietary carbohydrate levels diets.

It was found that glutathione (GSH) and total antioxidant capacity (T-AOC) showed a trend of decreasing with the dietary carbohydrate in the intestine. Peak contents of malondialdehyde (MDA) in the liver and intestines were recorded in the S12 group, showing significantly higher compared to S8. GSH of liver and intestine in S12 was significantly lower than that of S8 (*P* < 0.05), and there was no significant disparity between S8 and S10. The liver T-AOC of S12 was significantly lower than that of S10 (*P* < 0.05), and the intestine T-AOC of S12 was significantly lower than that of S8 (*P* < 0.05), and there was no significant disparity in the liver and intestinal T-AOC between S8 and S10 (Figure 3).

### 3.6. The mRNA Expression in Liver

**Figure 4:**
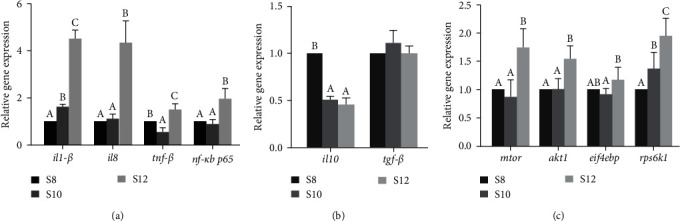
(a–c) The mRNA expression in liver of pikeperch fed different dietary carbohydrate levels diets.

The mRNA expression of *il1-β*, *il8*, and *nf-κb p65* increased with the increase of carbohydrate level and reached a significant maximum in S12. The *tnf-β* in S12 was significantly higher than that in S8 and S10 (*P* < 0.05; Figure 4(a)). The mRNA expression of *il10* was significantly higher in S8 than that in S10 and S12 (*P* < 0.05). The mRNA expression of *tgf-β* in the intestine was lower in S8 and S10 than in S12, but the difference was not significant (Figure 4(b)).

The study found that the mRNA expression of *mtor*, *akt1*, and *rps6k1* achieved a significant maximum in S12 (*P* < 0.05). The expression of *eif4ebp* reached the maximum in S12 and the minimum in S10 (Figure 4(c)).

### 3.7. The mRNA Expression in Intestine

**Figure 5:**
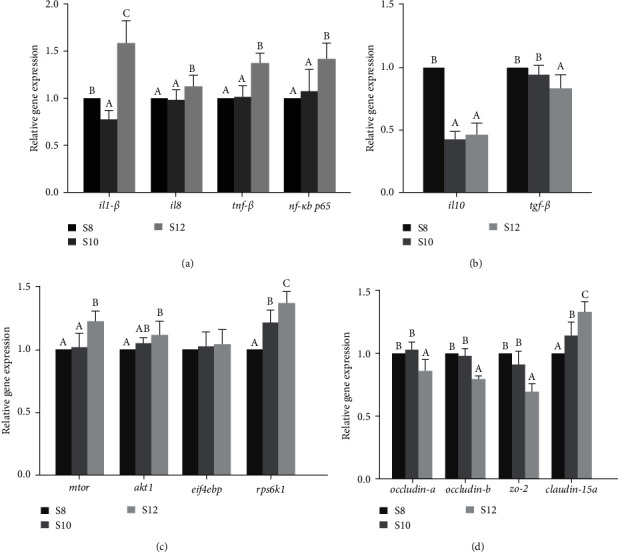
(a–d) The mRNA expression in intestine of pikeperch fed different dietary carbohydrate levels diets.

The *il1-β*, *il8*, *tnf-β*, and *nf-κb p65*, all achieved maximum mRNA expression at S12, with no significant difference in the expression of *il8* and *tnf-β* at S8 and S10 (*P* > 0.05; Figure 5(a)). The *il10* expression achieved a significant maximum at S8 and no significant disparity at S10 and S12. The *tgf-β* showed a significant decrease in expression with increasing levels of carbohydrate (*P* < 0.05; Figure 5(b)).

As dietary carbohydrate levels rose, there was an increase in the activity of genes linked to the mTOR pathway (*mtor*, *akt1*, *eif4ebp*, and *rps6k1*). However, all of them were significantly affected by dietary carbohydrate levels, except for the change in the expression of the *eif4ebp* was not significantly different (Figure 5(c)).

Downregulation of *occludin-a*, *occludin-b*, and *zo-2* in the intestine was observed in S12 compared with S8 and S10, whereas *claudin-15a*, in contrast, achieved maximum expression at S12, while there was no significant disparity between S8 and S10 for *occludin-a*, *occludin-b*, and *zo-2* (Figure 5(d)).

### 3.8. Intestinal Microflora

Diversity analysis was performed on three groups (five samples per group), resulting in 837,689 sequences averaging 417 bp in length. Each group's sequencing coverage surpassed 99%, with no significant disparity between groups (*P* > 0.05). The dilution curve neared saturation, indicating that the sequencing results can be further analyzed. Evaluating the *α*-diversity index reveals a decline in Sobs, Ace, and Chao correlating with higher levels of dietary carbohydrates (*P* < 0.05). Shannon showed a trend of first decreasing and then increasing, while Simpson and Shonnon showed the opposite trend (*Supplementary table 3*).

**Figure 6:**
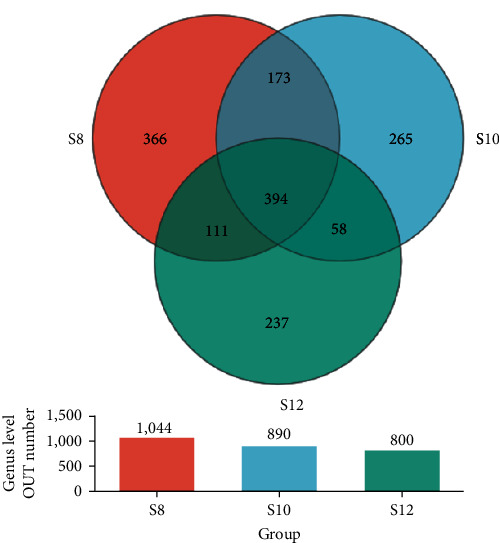
Veen diagram analysis of shared and unique OTUs between groups at the genus level.

**Figure 7:**
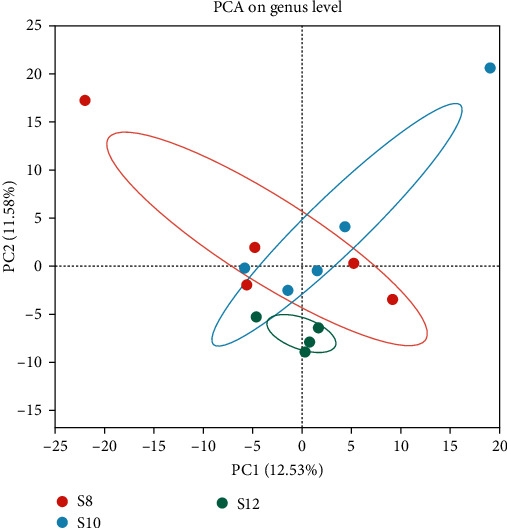
Effect of dietary carbohydrate on intestinal microflora communities of pikeperch by *β*-diversity measures.

**Figure 8:**
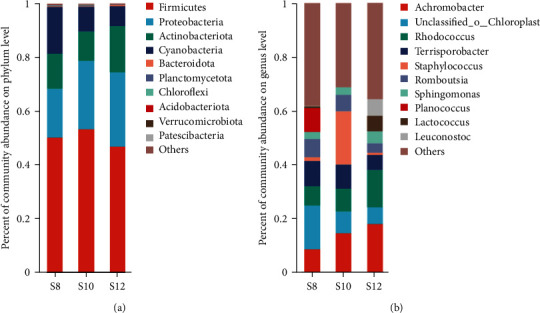
Effects of different dietary carbohydrate levels on relative abundance of intestinal microflora in juvenile pikeperch. (a) At the phylum level and (b) At the genus level.

**Figure 9:**
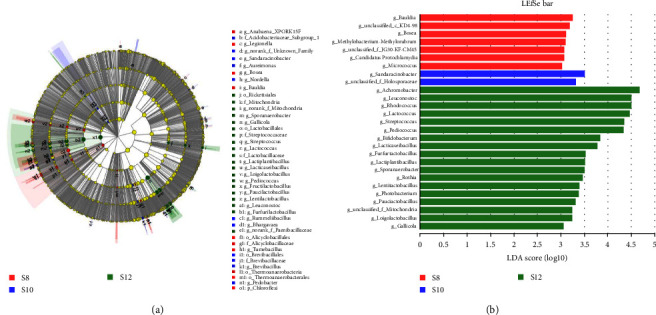
Intergroup variation in the relative abundance of intestinal microflora communities at the genus level. (a) Cladogram from LEfSe. (b) LDA score from LEfSe-PICRUSt.

The Veen chart showed that S8, S10, and S12 have 366, 265, and 237 unique OTUs, respectively, and there were 394 OTUs jointly owned between groups (Figure 6). The results of PCA analysis showed that S8 and S10 flora were similar at the genus level, while S12 was different from S8 and S10 (Figure 7). At the phylum level, with the increase of dietary carbohydrate levels, the number of Proteobacteria increased continuously, while Firmicutes increased first and then decreased (Figure 8(a)). At the genus level, *Achromobacter* and *Rhodococcus* increased with increasing dietary carbohydrate addition, while unclassified_o__Chloroplast and *Terrisporobacter* had the opposite trend (Figure 8(b)). LEfSe analysis revealed that the dominant bacteria genera in S8 were *Bauldia*, *Bosea*, *Methylobacterium*–*Methylorubrum*, and *Micrococcus*; the dominant bacteria genera in S10 was *Sandaracinobacter*; and the dominant bacteria genera in S12 were *Achromobacter*, *Leuconostoc*, *Rhodococcus*, *Lactococcus*, *Streptococcus*, and *Pediococcus* (Figure 9).

## 4. Discussion

For animals, carbohydrates represent the most affordable energy source in their diet, and it is necessary to increase dietary carbohydrate levels as much as possible to minimize the addition of protein [31]. Nevertheless, carbohydrate requirements are inconsistent from fish to fish. Thus, determining the optimum carbohydrate requirement for a specific cultured fish is of interest to feed producers and researchers. Our results showed that the growth performance (WGR and SGR) of S8 and S10 was significantly higher than that of S12, suggesting that pikeperch have the ability to utilize carbohydrates. Earlier research indicates that feeds with excessive carbohydrate levels can impair the growth of aquatic creatures [16]. The current research found a notable reduction in WGR and SGR as dietary carbohydrates rose in juvenile pikeperch, aligning with earlier findings in grouper (*Epinephelus akaara*) [16] and large yellow croaker (*Larimichthys crocea*) [32]. It indicated that excessive carbohydrate levels are detrimental to the growth of pikeperch. Findings from this research indicated that dietary carbohydrate levels had negatively influenced the growth performance of pikeperch, which was similar to the studies on largemouth bass (*Micropterus salmoides*) [33] and grouper [16].

It is widely acknowledged that the intestine serves as a crucial physical barrier for fish, and its structural soundness is vital for sustaining this barrier function [34, 35]. Findings from this research indicated that 12% starch levels of dietary carbohydrates had a negative effect on the intestinal mucosa of pikeperch, which was also observed in snakeheads (*Channa argus*) [36] and largemouth bass [20]. Excess carbohydrates create a chronic stress on the intestinal mucosa and damage the intestinal structure. In addition, the intestinal barrier is not only related to the integrity of the intestinal mucosa but also to the permeability of the intestinal epithelium [37]. Occludin, Claudins, and ZO are transmembrane proteins involved in maintaining tight junction stability and barrier function. Reduced expression of the *occludin* and *zo* genes and increased expression of the *claudin-15a* gene all result in abnormal intestinal permeability [38, 39, 40, 41]. In the present study, *occludin-a*, *occludin-b*, and *zo-2* genes were significantly downregulated, and the *claudin-15a* gene was significantly upregulated in the S12 group, indicating that 12% starch had a deleterious effect on pikeperch epithelial cells.

Research indicates that the amount of carbohydrates in the diet significantly influences intestinal digestion [42]. To a certain extent, the addition of appropriate levels of carbohydrates to the diet can enhance *α*-amylase activity, whereas excessive dietary carbohydrate levels will have a negative impact on the digestive and absorption function of the digestive tract [43, 44, 45]. The study found that pikeperch with varying carbohydrate intake had decreased intestinal *α*-amylase activity as carbohydrate levels in their feed increased. This outcome aligns with earlier studies conducted on rainbow trout (*Oncorhynchus mykiss*) [46] and Atlantic salmon (*Salmo salar*) [47]. High levels of carbohydrates can inhibit amylase due to the adsorption of raw starch to amylase, and the accelerated passage of starch through the intestine reduces the time of digestion and absorption in the intestine [46]. With the appropriate addition of carbohydrates, trypsin and lipase activities were elevated, leading to the lowest feed conversion rate at S10 (FCR = 0.93); when carbohydrate levels increased further, while trypsin and lipase declined with the consequent increase in feed conversion rate at S12 (FCR = 1.47). This reduction may be the adsorption of raw starch to trypsin and lipase, resulting in decreased enzyme activity. In addition, inhibition of trypsin and lipase activities by high dietary carbohydrates has been reported in previous studies [48, 49, 50].

Oxidative stress is a crucial defense mechanism for organisms. However, it is capable of generating dangerous compounds like superoxide anions, hydrogen peroxide, and hydroxyl radicals [51]. Cells have developed an antioxidant defense mechanism, incorporating antioxidant enzymes, to lessen the harm inflicted by these substances. By eliminating surplus reactive oxygen species (ROS) and hydroxyl radicals, the antioxidant defense mechanism shields cells and tissues from oxidative stress-induced harm [52, 53]. Endogenous antioxidants like SOD and CAT can neutralize ROS [35]. SOD, CAT, and T-AOC can be used to assess an organism's antioxidant capacity by nonenzyme or enzyme components [54, 55]. MDA, a product of lipid peroxidation, is mainly used as a marker of cell damage [56]. This study showed that SOD, CAT, and T-AOC significantly decreased, and MDA concentration significantly increased when the starch content in the diet was 12%, indicating that the intestinal antioxidant system was damaged at 12% starch level. This finding had similar results in blunt snout bream (*Megalobrama amblycephala*) [57] and largemouth bass [20]. However, the impact of carbohydrates on the antioxidant abilities of aquatic animals remains a matter of debate. In the study on golden pompano (*Trachinotus ovatus*) [35], it was indicated that antioxidant enzyme activity correlated with higher dietary carbohydrate intake. Furthermore, in the research on European sea bass (*Dicentrarchus labrax*) [58], it was noted that a high carbohydrate diet had no effect on antioxidant enzyme activity. Excessive carbohydrates lead to persistent high sugar levels in fish, which eventually lead to chronic stress [59]. Different aquatic animals have different tolerance to high sugar, which may be one of the reasons for this phenomenon. The root cause of this phenomenon needs more experiments to explore.

Cytokines play a crucial role as signaling entities that control immune cells, and their presence may indicate the condition of the body's immune reaction. Numerous studies have shown that carbohydrate diets induce an intestinal inflammatory response in fish [20, 60, 61]. The inflammatory response in the intestine is determined by a variety of cytokines involved [62]. Cytokines are usually divided into proinflammatory (*il1-β*, *il8*, and *tnf-β*) and anti-inflammatory (*il10* and *tgf-β*) factors [63, 64]. The results of the present study showed that *il1-β*, *il8*, and *tnf-β* expression levels were significantly increased, while *il10* and *tgf-β* expression levels were significantly decreased when pikeperch were fed a high carbohydrate diet. These results were also observed in largemouth bass and golden pompano [20, 65]. In aquatic animals, increased expression of *il8* and *il1-β* and decreased expression of *il10* can cause inflammatory responses and lead to tissue damage [60, 66]. Previous studies have found that high dietary carbohydrates can cause excessive accumulation of liver glycogen in carnivorous fish, resulting in liver cell damage [61, 67, 68]. In this study, the expression of *il8* and *il1-β* was increased, and the expression of *il10* was decreased, indicating that 12% starch supplementation would lead to impaired liver found iin pikeperch. The cause of this occurrence could be the stimulation of the *nf-κb p65* gene by elevated glucose levels, which in turn triggers the generation of cytokines like *il1-β*, *il8*, and *il10*, culminating in the organism's inflammatory reaction [69, 70, 71].

The *mTOR* belongs to a serine/threonine protein kinase member of the phosphoinositide-3-kinase (PI3K) family [72, 73]. This entity merges with various proteins to create two distinct complexes, named mTORC1 and mTORC2, each with unique roles [74, 75]. The regulation of mTORC1 is influenced by a range of environmental and cellular factors, including stress, growth factors, and energy levels, and it stimulates protein and lipid synthesis [72, 76, 77]. mTORC1 targets two key elements: p70 S6 kinase (*S6K*) and eukaryotic initiation factor 4E (*eif4e*)-binding protein-1 (*4e-bp1*), both of which regulate mRNA translation [74, 78]. A previous study found that inhibition of mTORC1 blocked adipogenesis and impaired adipocyte maintenance [79, 80, 81], while over activating mTORC1 promoted adipogenesis [82]. This study found that high levels of starch addition activated mTOR-related genes, indicating that adipogenesis was stimulated at high energy levels. This could explain the occurrence of liver lipid deposition at high starch levels, resulting in an increase in HSI and VSI. However, this aspect of the analysis did not clearly explain why the decreased weight gain rate of the fish happened at high starch levels. We hypothesized that the low ratio of protein to energy may be the cause. Although this ratio met the energy requirement for fish growth, it did not provide enough amino acids. Similar results were found in previous studies where the low ratio protein to energy inhibited fish growth [83, 84].

The effect of dietary starch level on the intestinal microflora of juvenile pikeperch was reported for the first time in this study. In this study, it could be found that feed starch level can change the intestinal microflora of juvenile pikeperch. With the increase of feed starch level, the abundance of intestinal microflora gradually decreased, while the diversity is not linearly related to feed starch content. Cluster analysis results revealed that S12 was separated from S8 and S10. While the *α*-diversity index may indicate the intestinal microflora's ecological role, its advantageous effects frequently depend on the richness of advantageous species [85]. Firmicutes, Proteobacteria, and Actinobacteriota are major phylum in the intestinal microflora of most farmed fishes [86, 87]. The Firmicutes phylum is a group of anti-inflammatory bacteria [87], while the Proteobacteria and Actinobacteriota include many pathogenic bacteria [88, 89], the abundance of which correlates with the status of intestinal inflammation. The data suggested that higher dietary starch levels led to a decrease in Firmicutes abundance and an increase in Proteobacteria and Actinobacteriota abundance, which might be an important reason why intestinal inflammation occurs in the intestine on a diet with high starch levels. At the genus level, incorporating starch into the diet led to an increase in the abundance of *Achromobacter* and *Rhodococcus*. *Achromobacter* and *Rhodococcus* are frequently found in water and soil and are known digestive pathogens [88, 90, 91]. A lack of nutrients and physiological dysfunctions heighten the risk of pathogenic bacteria when the water environment deteriorates. The results suggest that a 12% starch level might increase the probability of pikeperch infection with *Achromobacter* and *Rhodococcus*. The other attention point, *Lactococcus*, as a probiotic, was elevated in S12 comparison to the other two groups. The reason may be that the carbohydrate level of S12 was too high, some carbohydrates were not digested and absorbed and accumulated in the intestine, which become a nutrient source for *Lactococcus*, resulting in a large number of *Lactococcus* in the intestine.

## 5. Conclusion

In the present study, juvenile pikeperch fed a dietary starch should be 8% based on the growth performance, liver, and intestine immune. The dietary starch level can be increased to 10% when both growth performance and feed cost are considered. Additionally, elevating the levels of carbohydrates in the diet had a protein-sparing effect in pikeperch.

## Figures and Tables

**Table 1:** The formulation and composition of experimental diets.

Item	S8	S10	S12
Ingredients (%)			
Fish meal	48.00	48.00	48.00
Poultry meal	8.00	8.00	8.00
Corn gluten meal	7.00	7.00	7.00
Soy protein concentrate	10.88	10.88	10.88
Tapioca starch^a^	8.00	10.00	12.00
Fish oil	4.50	4.50	4.50
Lecithin oil	2.00	2.00	2.00
Premix-V + M^b^	1.00	1.00	1.00
Choline chloride	0.50	0.50	0.50
Cellulose	9.00	7.00	5.00
Marker (Yi_2_O_3_)	0.10	0.10	0.10
Binder	1.00	1.00	1.00
BHT	0.02	0.02	0.02
Chemical composition (percentage of dry matter)			
Moisture	4.15	4.21	4.22
Crude protein	51.05	51.04	50.97
Crude lipid	11.09	11.15	11.14
Carbohydrates	15.17	17.12	19.05
Ash	11.90	11.79	11.85
Gross energy^c^ (kJ/g)	19.04	19.39	19.71

^a^Compared with cereal starch, tapioca starch contains more branched-chain starch that is favorable for fish digestion and has better viscosity, penetration, film-forming properties, etc. The crude protein and crude ash contents are lower than those of corn starch, and it has better physicochemical properties and is widely used in many kinds of fish [21, 22, 23], so tapioca starch was selected as a source of feed starch in this study. ^b^Premix-V + M (mg/kg or IU/kg diet): VA, 750,000 IU; VD_3_, 200,000 IU; VE, 6,000 mg; VK_3_, 2,000 mg; VB_1_, 1,200 mg; VB_2_, 1,200 mg; VB_12_, 8 mg; VC, 21,000 mg; D-calcium pantothenate, 2,000 mg; niacinamide, 9,000 mg; folic acid, 370 mg; D-biotin, 15 mg; inositol, 10,000 mg; MgSO_4_, 6,000 mg; ZnSO_4_, 4,000 mg; MnSO_4_, 2,500 mg; CuSO_4_, 2,500 mg; FeSO_4_, 2,500 mg; CoSO_4_, 160 mg; Ca(IO_3_)_2_, 200 mg; and Na_2_SeO_3_, 40 mg. ^c^Calculated using the mean values for carbohydrates (17.2 kJ/g), proteins (23.6 kJ/g), and lipids (39.5 kJ/g) according to NRC (2011).

**Table 2:** Growth performance of pikeperch fed diets with varying dietary carbohydrate levels.

Item	S8	S10	S12
IBW (g)	1.20 ± 0.01	1.19 ± 0.01	1.20 ± 0.01
FBW (g)	10.17 ± 0.11^b^	9.79 ± 0.39^b^	7.77 ± 0.35^a^
SR (%)	80.84 ± 7.29^b^	89.80 ± 1.10^b^	62.50 ± 3.21^a^
WGR (%)	748.13 ± 11.09^b^	716.84 ± 37.83^b^	548.07 ± 32.60^a^
SGR (%/day)	3.82 ± 0.02^b^	3.75 ± 0.08^b^	3.33 ± 0.09^a^
FCR	0.96 ± 0.05^a^	0.93 ± 0.01^a^	1.47 ± 0.01^b^
CF (g/cm^3^)	0.94 ± 0.03^b^	0.84 ± 0.06^b^	0.68 ± 0.03^a^
VSI (%)	14.20 ± 2.25	15.44 ± 0.94	17.52 ± 0.86
HSI (%)	1.63 ± 0.13^a^	1.36 ± 0.03^a^	2.25 ± 0.23^b^

*Note*: Values with different superscripts in the same row are significantly different (*P* < 0.05). IBW means the initial body weight (g), FBW means the final body weight (g), SR means the survival rate (%), WGR means the weight gain rate (%), SGR means the specific growth rate (%/day), FCR means the feed conversion rate (%), CF means the condition factor (g/cm^3^), VSI means the viscerosomatic index (%), HSI means the hepatosomatic index (%).

**Table 3:** Proximate composition of whole fish fed different diets.

Treatment	Moisture	Crude protein	Crude lipid	Ash
S8	72.63 ± 0.71	17.72 ± 0.46^b^	6.54 ± 0.24^b^	3.40 ± 0.10
S10	72.83 ± 0.22	17.52 ± 0.06^b^	6.44 ± 0.10^b^	3.42 ± 0.03
S12	73.80 ± 0.89	15.07 ± 0.05^a^	5.46 ± 0.32^a^	3.50 ± 0.01

*Note*: Label differences superscripts in the same column indicate significant differences (*P* < 0.05).

**Table 4:** Effect of different dietary carbohydrate levels on midgut morphological analysis of pikeperch.

Treatment	Villus height	Villus width	Intestinal wall thickness
S8	344.66 ± 7.57^c^	74.44 ± 8.03^b^	69.13 ± 2.03^b^
S10	308.57 ± 5.58^b^	59.64 ± 3.23^ab^	65.79 ± 2.05^b^
S12	234.30 ± 12.23^a^	46.16 ± 3.41^a^	56.15 ± 0.61^a^

*Note*: Label differences superscripts in the same column indicate significant differences (*P* < 0.05).

## Data Availability

The data that support the findings of this study are available from the corresponding author upon reasonable request.

## Supplementary Materials

Supplementary 1 Table 1: material and methods.

Supplementary 2 Table 2: specific primers for real-time PCR.

Supplementary 3 Table 3: intestinal microflora diversity of pikeperch.
