# Supplementary material for: Effects of Different Levels of Carbohydrates on Growth Performance, Hepatic and Intestinal Health, and Intestinal Microflora of Juvenile Pikeperch (Sander lucioperca)
Source: Aquac Nutr. 2024 Aug 9;2024:8450154. doi: 10.1155/2024/8450154 (PMC11329307; doi:10.1155/2024/8450154)
Supplement: Supplementary 1 — Table 1: material and methods. [file 8450154.f1.docx]

Table S1 Material and methods

|  | Item | Description | Method or reagent source |
| --- | --- | --- | --- |
| Chemical composition of whole fish | Moisture | Moisture | GB/T6435-2014 |
|  | Crude protein | Crude protein | GB/T6432-1994 |
|  | Crude lipid | Crude lipid | GB/T6433-2006 |
|  | Ash | Ash | GB/T6438-2007 |
| Antioxidant enzyme and digestive enzyme activity | SOD | Superoxide Dismutase | Commercial kits (Jiancheng Biotech. Co., Nanjing, China) |
|  | GSH | Glutathione |  |
|  | MDA | Malondialdehyde |  |
|  | T-AOC | Total antioxidant capacity |  |
|  | AMS | α-amylase |  |
|  | Trypsin | Trypsin |  |
|  | LPS | Lipase |  |
| Gene expression | *il1-β* | Interleukin 1 beta | Takara TB Green Premix Ex Taq II (Tli RnaseH Plus) |
|  | *il8* | Interleukin 8 |  |
|  | *il10* | Interleukin 10 |  |
|  | *nf-κb p65* | Nuclear transcription factor-κB P65 |  |
|  | *akt1* | AKT serine/threonine kinase 1 |  |
|  | *mtor* | Mechanistic target of rapamycin kinase |  |
|  | *eif4ebp* | Eukaryotic initiation factor 4E binding protein |  |
|  | *rps6k1* | Ribosomal protein S6 kinase B1 |  |
|  | *clandin-15a* | Clandin-15a |  |
|  | *occluding-a* | Occluding-a |  |
|  | *occluding-b* | Occluding-b |  |
|  | *zo-2* | Zonula occludens-2 |  |
|  | *tnf-β* | Tumor necrosis factor beta |  |
|  | *tgf-β* | Transforming growth factor beta |  |
