# Supplementary material for: Effects of Different Levels of Carbohydrates on Growth Performance, Hepatic and Intestinal Health, and Intestinal Microflora of Juvenile Pikeperch (Sander lucioperca)
Source: Aquac Nutr. 2024 Aug 9;2024:8450154. doi: 10.1155/2024/8450154 (PMC11329307; doi:10.1155/2024/8450154)
Supplement: Supplementary 2 — Table 2: specific primers for real-time PCR. [file 8450154.f2.docx]

Table S2 Specific primers for Real-time PCR

| Target gene |  | Primer sequence (5'→3') | Accession number |
| --- | --- | --- | --- |
| *gapdh* | F | GGACCCATGAAGGGCATTCT | XM_031299603 |
|  | R | TGGCATAGTGCAGTGACGAG |  |
| *il1-β* | F | TGCAGTCTGTGGCTAACCTG | XM_031287947 |
|  | R | TTCTCTTCACGCCTGTGGAC |  |
| *il8* | F | GATGAGTCTGAGAAGCCTGGG | XM_031286001.2 |
|  | R | TCTCGTCGCAATGAGAGTTGG |  |
| *il10* | F | AACCAATGCTGTCGTTTCGTG | XM_031323076 |
|  | R | CATTGGTCTCCTCCGTCACTC |  |
| *nf-κb p65* | F | AACATGGCTACTACGAGGCG | XM_031279491.2 |
|  | R | TTCGCCTCGGGAATGTTGAA |  |
| *akt1* | F | GAGATGATGTGCGGCAGACT | XM_031279117.2 |
|  | R | CCGAGGAAAGCGGATGTCTT |  |
| *mtor* | F | CCATCCTCATCGGCCATTCA | XM_031286196.2 |
|  | R | TGCCTCTGGACAGGGAAATG |  |
| *occludin-a* | F | ATCATCTGCGCCATCCTAGC | XM_035991813.1 |
|  | R | TCCAGGACGCAGTAGTGGTA |  |
| *occludin-b* | F | ATGGATCCCAAAGCTGGCAA | XM_031300915.2 |
|  | R | AACTTTGATGAGCGGGCAGT |  |
| *zo-2* | F | ACTGGCCTCTTATCCGAGCA | XM_031300456.2 |
|  | R | CAGCACGTCTGACACGATGA |  |
| *rps6k1* | F | CCATTGCCCTCTCAGGGATG | XM_031278705.1 |
|  | R | GACTCAGCAATTCGCAAGCC |  |
| *claudin-15a* | F | CGAACCGTTACTGGAGGACC | XM_031289825.2 |
|  | R | AAGGCACGAGACGCTTGAAT |  |
| *eif4ebp* | F | TCGTCAGTTTAGCGAGAGCA | XM_031306363.2 |
|  | R | TGGGGCAGTCTGAGCAATAG |  |
| *tnf-β* | F | TGGCCCTTTGTTTAGGAGGC | XM_031313322.2 |
|  | R | GTCTGGCCTGGTTGTGTCAT |  |
| *tgf-**β* | F | TTTTGGCCCTGTACCAGCAT | XM_031285154.2 |
|  | R | GCCTGCCCACGTAATAGAGG |  |
