# Supplementary material for: Effects of Different Levels of Carbohydrates on Growth Performance, Hepatic and Intestinal Health, and Intestinal Microflora of Juvenile Pikeperch (Sander lucioperca)
Source: Aquac Nutr. 2024 Aug 9;2024:8450154. doi: 10.1155/2024/8450154 (PMC11329307; doi:10.1155/2024/8450154)
Supplement: Supplementary 3 — Table 3: intestinal microflora diversity of pikeperch. [file 8450154.f3.docx]

Table S3 Intestinal microflora diversity of pikeperch

| Items | Sobs | Shannon | Simpson | Ace | Chao | Coverage |
| --- | --- | --- | --- | --- | --- | --- |
| S8 | 216.83±9.41^b^ | 2.92±0.17 | 0.13±0.03 | 222.59±10.62^b^ | 224.49±12.14 | 0.999±0.000 |
| S10 | 179.33±17.14^a^ | 2.64±0.31 | 0.20±0.08 | 184.26±17.45^a^ | 187.05±17.81 | 0.999±0.000 |
| S12 | 173.83±5.29^a^ | 3.10±0.91 | 0.09±0.01 | 181.50±5.81^a^ | 185.01±6.48 | 0.999±0.000 |

Note: label differences superscripts in the same column indicate significant differences (*P*<0.05).
